# Sepsis is associated with reduced spontaneous neutrophil migration velocity in human adults

**DOI:** 10.1371/journal.pone.0205327

**Published:** 2018-10-09

**Authors:** Steven L. Raymond, Russell B. Hawkins, Julie A. Stortz, Tyler J. Murphy, Ricardo Ungaro, Marvin L. Dirain, Dina C. Nacionales, McKenzie K. Hollen, Jaimar C. Rincon, Shawn D. Larson, Scott C. Brakenridge, Frederick A. Moore, Daniel Irimia, Phil A. Efron, Lyle L. Moldawer

**Affiliations:** 1 Department of Surgery, University of Florida College of Medicine, Gainesville, Florida, United States of America; 2 Massachusetts General Hospital and Harvard Medical School, Boston, Massachusetts, United States of America; Medical College of Georgia, Augusta, UNITED STATES

## Abstract

Sepsis is a common and deadly complication among trauma and surgical patients. Neutrophils must mobilize to the site of infection to initiate an immediate immune response. To quantify the velocity of spontaneous migrating blood neutrophils, we utilized novel microfluidic approaches on whole blood samples from septic and healthy individuals. A prospective study at a level 1 trauma and tertiary care center was performed with peripheral blood samples collected at <12 hours, 4 days, and/or 14 days relative to study initiation. Blood samples were also collected from healthy subjects. *Ex vivo* spontaneous neutrophil migration was measured on 2 μl of whole blood using microfluidic devices and time-lapse imaging. For each sample, individual neutrophils were tracked to calculate mean instantaneous velocity. Forty blood samples were collected from 33 patients with sepsis, and 15 blood samples were collected from age- and gender-matched healthy, control subjects. Average age was 61 years for septic patients with a male predominance (67%). Overall, average spontaneous neutrophil migration velocity in septic samples was 16.9 μm/min, significantly lower than controls samples at 21.1 μm/min (p = 0.0135). Neutrophil velocity was reduced the greatest at <12 hours after sepsis (14.5 μm/min). Regression analysis demonstrated a significant, positive correlation between neutrophil velocity and days after sepsis (p = 0.0059). There was no significant association between neutrophil velocity and age, gender, APACHE II score, SOFA score, sepsis severity, total white blood cell count, or percentage of neutrophils. Circulating levels of the cytokines IL-6, IL-8, IL-10, MCP-1, IP-10, and TNF were additionally measured using bead-based multiplex assay and found to peak at <12 hours and be significantly increased in patients with sepsis at all three time points (<12 hours, 4 days, and 14 days after sepsis) compared to healthy subjects. In conclusion, these findings may demonstrate an impaired ability of neutrophils to respond to sites of infection during the proinflammatory phase of sepsis.

## Introduction

Sepsis is a common and serious complication among trauma and surgical patients, especially among those admitted to the intensive care unit (ICU) [1–4]. Approximately 160,700 cases of severe sepsis occur in surgical patients every year in the United States [1]. Thirty-day mortality rates of general surgery patients with sepsis and septic shock are 5% and 34%, respectively [2]. Neutrophils are responsible for initiating an immediate immune response to invading pathogen by mobilizing to the site of infection. Animal studies have revealed that impairment of neutrophil migration is a crucial event in sepsis and correlates with sepsis outcomes [5–7]. Similarly, circulating neutrophils obtained from patients with sepsis exhibit less chemotactic activity compared to neutrophils from healthy volunteers [8–10]. Blood samples from patients with sepsis have additionally demonstrated unique neutrophil motility phenotypes compared to non-infected samples [11, 12]. This spontaneous motility phenotype can be quantified into a sepsis score based on observations from a drop of blood within novel microfluidic devices and can accurately segregate patients with sepsis from those without sepsis [12]. To quantify the velocity of individual spontaneous migrating neutrophils, we utilized these novel microfluidic approaches on whole blood samples from surgical and trauma patients admitted to the surgical intensive care unit (SICU) who had developed sepsis, and from healthy control subjects. We hypothesized that blood samples from septic patients will demonstrate reduced spontaneous neutrophil migration velocity compared to age- and gender-matched healthy controls, especially during the initial *‘cytokine storm’*, and that this reduction in velocity will return to control values as the proinflammatory surge resolves.

## Methods

### Study design

A prospective observational study was conducted between 2014 and 2018 at UF Health Shands Hospital, a 996-bed level 1 trauma and tertiary care center in Gainesville, Florida, USA (NCT: 02276417) [13]. The aim of the parent study was to examine the epidemiology, natural history, and long-term outcomes of surgical or trauma patients with sepsis. Blood samples were collected from patients at various time points including <12 hours, 4 days, and 14 days relative to sepsis protocol and study initiation. The early time point was chosen to examine whether biomarkers obtained in the first 24 hours could predict clinical trajectories and outcomes, whereas the two week time point was chosen to examine any temporal resolution in those patients who remained hospitalized at least two weeks and could be classified as having chronic critical illness [14]. Screening for sepsis was carried out using the Modified Early Warning Signs-Sepsis Recognition System (MEWS-SRS) [15]. All enrolled patients were managed using a standardized sepsis protocol based on the Surviving Sepsis Consensus guidelines. Initial treatment included intravenous fluid administration and initiation of broad-spectrum antibiotics, which were later narrowed based on culture and sensitivity data.

### Inclusion/Exclusion criteria

Patients eligible for participation in the study met the following inclusion criteria: (1) age ≥18 years; (2) presence in the SICU, where clinical care was protocolized and provided by the critical care team; and (3) clinical diagnosis of sepsis, severe sepsis, or septic shock as defined by the 2001 SCCM/ESICM/ACCP/ATS/SIS International Sepsis Definitions Conference [16]. Sepsis was clinically diagnosed by the bedside physician given the relatively high rate of culture negative sepsis reported in the literature [17]. All patients with sepsis were then clinically adjudicated in a prospective fashion by the investigators at weekly program adjudication meetings.

Patients were excluded if any of the following were present: (1) refractory shock; (2) uncontrollable sepsis with an inability to achieve source control; (3) pre-sepsis expected lifespan <3 months; (4) patient/family not committed to aggressive management; (5) severe congestive heart failure (CHF) (NYHA Class IV); (6) Child-Pugh Class C liver disease or pre-liver transplant; (7) known human immunodeficiency virus (HIV) with CD4^+^ count <200 cells/mm^3^; (8) patients receiving chronic corticosteroids or immunosuppressive agents, including organ transplant recipients; (9) pregnancy; (10) institutionalized patients; (11) inability to obtain informed consent; (12) chemotherapy or radiotherapy within 30 days; (13) severe traumatic brain injury; or (14) spinal cord injury resulting in permanent sensory and/or motor deficits.

### Enrollment and consent

This study was approved by the University of Florida Institutional Review Board (IRB) prior to initiation. A 96-hour waiver of informed consent was granted under existing precedent by the IRB in order to allow initial blood sampling and data collection in this critically ill population prior to obtaining consent, which was then obtained after the patient regained consent ability, or after contact and consent from the designated patient proxy. The IRB considered potential study subjects to be a ‘vulnerable’ population at time of admission, and the purpose of delayed consent was to permit the study patients or their legally assigned representative sufficient time to understand the nature, risks and benefits, and requirements of the study. In all subjects, signed informed consent was obtained from the patient or their legally assigned representative within 96 hours of study enrollment.

### Healthy control subjects

Healthy control subjects who were age- and gender-matched to the sepsis population were studied. Control subjects denied the following: (1) illness/infection within the past 2 weeks; (2) hospitalization within the last 2 weeks; (3) pregnancy; or (4) history of chronic corticosteroid or immunosuppressive use. Signed informed consent was obtained prior to blood sampling.

### Laboratory analyses

Peripheral ethylenediaminetetraacetic acid (EDTA) anticoagulated blood samples were collected, delivered on ice within 30 minutes, processed, and analyzed for spontaneous neutrophil migration velocities and proinflammatory cytokine concentrations for healthy controls once and for septic patients at the following time points relative to sepsis protocol and study initiation: <12 hours, 4 days, and 14 days.

Spontaneous neutrophil migration assay was performed with novel microfluidic devices which circumvent the need for neutrophil purification and preserve the physiological milieu. Microfluidic devices consist of a single whole blood loading chamber (WBLC) and eight migration chambers containing erythrocyte filters, migration channels, and mazes [12]. Erythrocyte filters block the dispersion of erythrocytes into the migration channel, whereas spontaneously migrating neutrophils can travel through without a decrease in speed. Erythrocyte filters additionally prevent the entrance of other, non-neutrophil leukocytes into the migration channels with neutrophils consisting of greater than 96% of cells entering the channels. Mazes located at the end of the migration channels allow neutrophils to change direction and continue spontaneous movement. The microfluidic devise were fabricated by BioMEMS Resource Center, Charlestown, Massachusetts. The scientific community can obtain the microfluidic device at cost by contacting the center directly (http://www.biomemsrc.org/about-bmrc/how-to-collaborate).

Microfluidic devices were primed with 200 μl of Hank’s Balanced Salt Solution (HBSS) with bovine albumin [final concentration 0.2 mg/ml] (Sigma-Aldrich, St. Louis, MO, USA) and then placed in a desiccator under vacuum for 10 minutes. The devices were then removed and allowed to equilibrate for at least 10 minutes until all channels had filled. HBSS with bovine albumin was then added to the well containing the device until the device was completely covered. Two microliters of whole blood stained with Hoechst Stain Solution (Sigma-Aldrich, St. Louis, MO, USA) were then pipetted into the WBLC using a gel-loading tip. Time-lapse imaging were performed on a Nikon Bio Station IMq^™^ microscope inside of a biochamber maintained at 37°C with 5% CO_2_. Each microfluidic device provided nine fields of view, one whole blood loading chamber and eight migration chambers containing the erythrocyte filters, migration channels, and mazes. Each field was imaged every 30 seconds to enable accurate tracking of cell movement, for 10 hours. Neutrophils were tracked and velocities calculated using ImageJ software (NIH, Bethesda, MD, USA) [18].

Plasma cytokine concentrations were measured by first centrifuging the blood at 1800 x g for 10 minutes at 4°C. Cell-free plasma was then collected and stored at -80°C until batch processing to reduce the degree of intra-assay variability. The concentrations of interleukin (IL)-6, IL-8 (CXCL8), IL-10, monocyte chemotactic protein-1 (MCP-1, CCL2), interferon-induced protein-10 (IP-10, CXCL10), and tumor necrosis factor (TNF) were determined using Milliplex Multiplex kits (EMD Millipore, Billerica, MA, USA) on the Luminex MAGpix Multiplex reader. Each sample was run in duplicate.

### Statistical analyses

Data are presented as either frequency and percentage, median and percentile, or mean and standard deviation (SD). Analysis of Variance (ANOVA) and student’s t-tests were used for comparison of continuous parametric variables. Kruskal-Wallis and Mann-Whitney tests were used for comparison of nonparametric variables. Correlations were calculated using Pearson’s correlation coefficient and linear regression analyses. Because plasma cytokine concentrations are not routinely normally distributed, all concentrations were log-transformed prior to analysis. All significance tests were two-sided, with p-values less than 0.05 considered statistically significant. Statistical analyses were performed with SAS software (Cary, NC, USA) and Prism GraphPad Software (La Jolla, CA, USA).

## Results

### Clinical characteristics and outcomes

Forty blood samples were collected from 33 patients with sepsis, and 15 blood samples were collected from 15 age- and gender-matched non-infected healthy, control subjects. One patient had blood samples for all three time points, 5 patients had blood samples for two time points, and 27 patients had blood samples for only one time point. Blood samples were unable to collect at all time points for all patients given patient factors and/or resource limitations. In addition, all 33 sepsis subjects met Sepsis-3 criteria for sepsis or septic shock. Average age was 61 years for septic patients (range 24–82) with a male predominance (67%) (Table 1). Of the 33 septic patients, 9 (27%) were diagnosed with sepsis, 14 (42%) with severe sepsis, and 10 (30%) with septic shock. The primary source of sepsis was intra-abdominal sepsis (33%), followed by surgical site (15%) and urinary tract (15%) infections. Median hospital and ICU length of stay was 17 and 9 days, respectively (Table 2). Median days on mechanical ventilation was five days. Median maximum Sequential Organ Failure Assessment (SOFA) score among septic patients was 9. Overall in-hospital mortality for this sepsis cohort was 18%.

**Table 1:** Baseline characteristics of septic adults.

Characteristics	Septic (n = 33)	Controls (n = 15)
Male, n (%)	22 (66.67)	9 (60.00)
Age in years, mean (SD)	61 (15)	60 (15)
Age ≥65 years, n (%)	17 (52)	6 (40)
BMI, median (25th, 75th)	28.4 (24.0, 36.0)	28.1 (22.5, 30.4)
Number of comorbidities,†, n (%)		
0	11 (33.33)	10 (66.67)
1	6 (18.18)	2 (13.33)
2	10 (30.30)	3 (20)
≥3	6 (18.18)	0 (0)
Charlson comorbidity index score, mean (SD)	3.25 (2.65)	2.47 (2.13)
APACHE II score, mean (SD)	19.6 (8.7)	
Delayed sepsis onset,‡, n (%)	12 (36)	
Inter-facility hospital transfer, n (%)	11 (33)	
Sepsis severity, n (%)		
Sepsis	9 (27)	
Severe sepsis	14 (42)	
Septic shock	10 (30)	
Sepsis diagnosis, n (%)		
Intra-abdominal sepsis	11 (33)	
Surgical site infection	5 (15)	
Urinary tract infection	5 (15)	
Pneumonia	4 (12)	
NSTI	4 (12)	
Other	4 (12)	

^†^Comorbidities included those defined within the Charlson Comorbidity Index.

^‡^Delayed sepsis onset, defined as sepsis occurring >2 days after hospital admission due to postsurgical or posttrauma infection.

**Table 2:** Outcomes of septic adults.

Outcomes	Septic (n = 33)
Hospital mortality, n (%)	6 (18)
ICU LOS, median (25th, 75th)	9 (3, 16)
Hospital LOS, median (25th, 75th)	17 (8, 27)
Ventilator days, median (25th, 75th)	5 (3, 15)
Maximum SOFA score^†^, median (25th, 75th)	9 (4, 12)
Multiple organ failure^‡^, n (%)	20 (61)
Discharge disposition, n (%)	
“Good” Disposition	21 (64)
Home	6 (18)
Rehab	3 (9)
Home healthcare services	12 (36)
“Poor” Disposition	12 (36)
Long Term Acute Care facility	5 (15)
Skilled Nursing facility	1 (3)
Another Hospital	0 (0)
Hospice	0 (0)
Death	6 (18)

^†^SOFA score, Composite score of 6 different organ systems (respiratory, cardiovascular, hepatic, coagulation, renal, and neurological systems) used to determine the extent of a patient’s organ dysfunction.

^‡^Multiple organ failure, SOFA component score ≥3 in at least 2 organs systems.

### Neutrophil velocities

**Fig 1:**
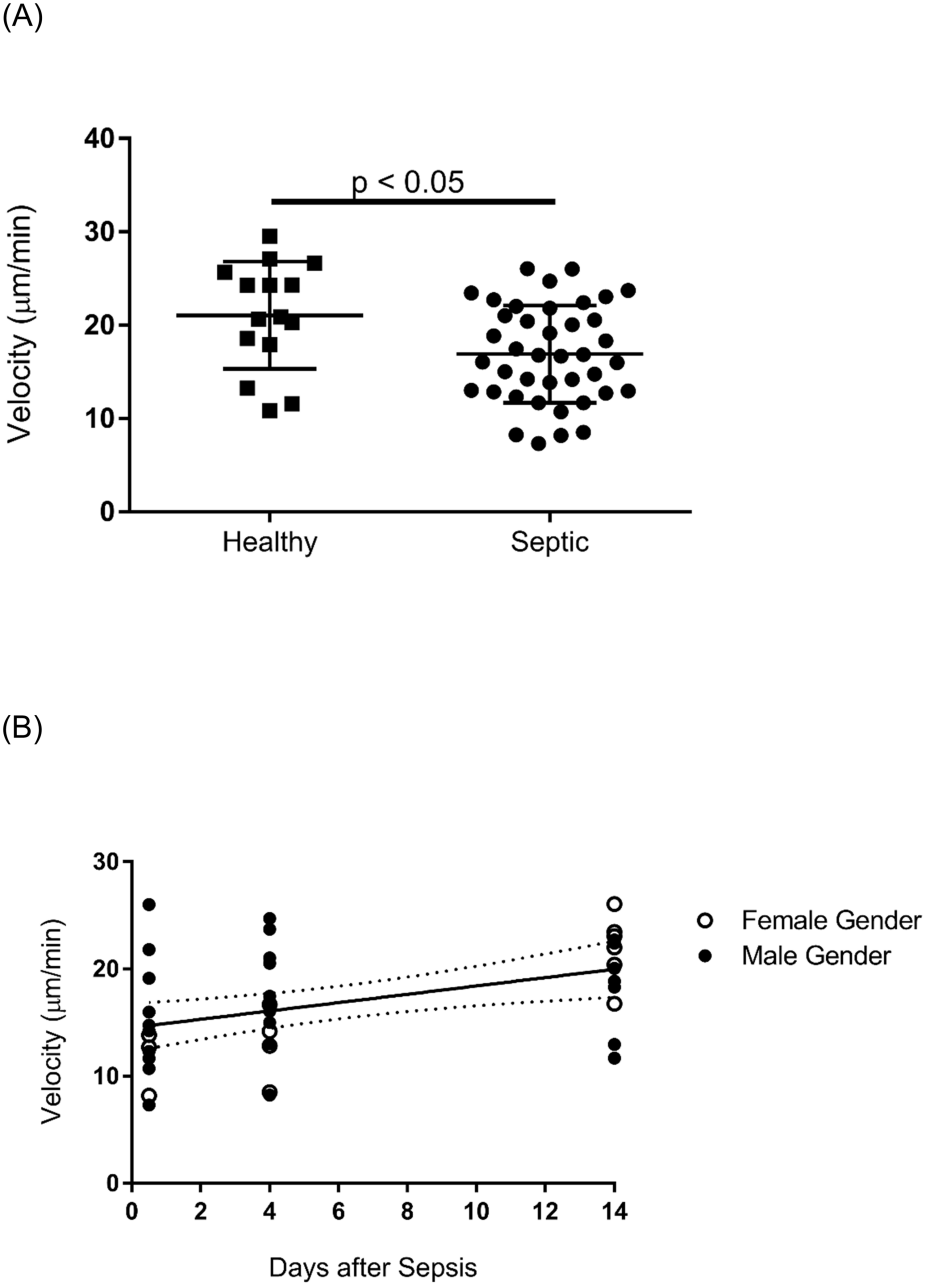
**(A)** The average spontaneous neutrophil migration velocity in septic samples was 16.9 μm/min, significantly lower than age-matched controls (21.1 μm/min; p = 0.014). n = 15 for healthy group, n = 40 for septic group. Data are means ± SD. **(B)** Neutrophil velocity had a significant, positive correlation with days after sepsis (r = 0.4281, p = 0.0059). Data are best-fit line with 95% confidence bands.

Average spontaneous neutrophil migration velocity for healthy control subjects was 21.1 μm/min. There was no significant difference in the velocity of spontaneously migrating neutrophils between healthy females and males (females: 24.1, males: 19.0 μm/min, p = 0.0905). Across the entire data set, the average spontaneous neutrophil migration velocity in septic samples was 16.9 μm/min, significantly lower than age- and gender-matched controls (p = 0.0135) (Fig 1A). Neutrophil velocity was reduced the greatest at less than 12 hours after sepsis (14.5 μm/min). Regression analysis demonstrated a significant, positive correlation between neutrophil velocity and days after sepsis (r = 0.4281, p = 0.0059) (Fig 1B). Neutrophil velocity 14 days after sepsis (19.9 μm/min) was not significantly different than healthy controls (21.1 μm/min, p = 0.5559). There was no significant difference in neutrophil velocity by sepsis severity (sepsis: 17.6, severe sepsis: 17.4, septic shock: 15.6 μm/min, p = 0.5873). Additionally, there were no significant correlations between neutrophil velocity and patient age, gender, Acute Physiology and Chronic Health Evaluation II (APACHE II) score, SOFA score, total white blood cell count, or percentage of neutrophils in the white blood cell population.

### Cytokine levels

**Fig 2:**
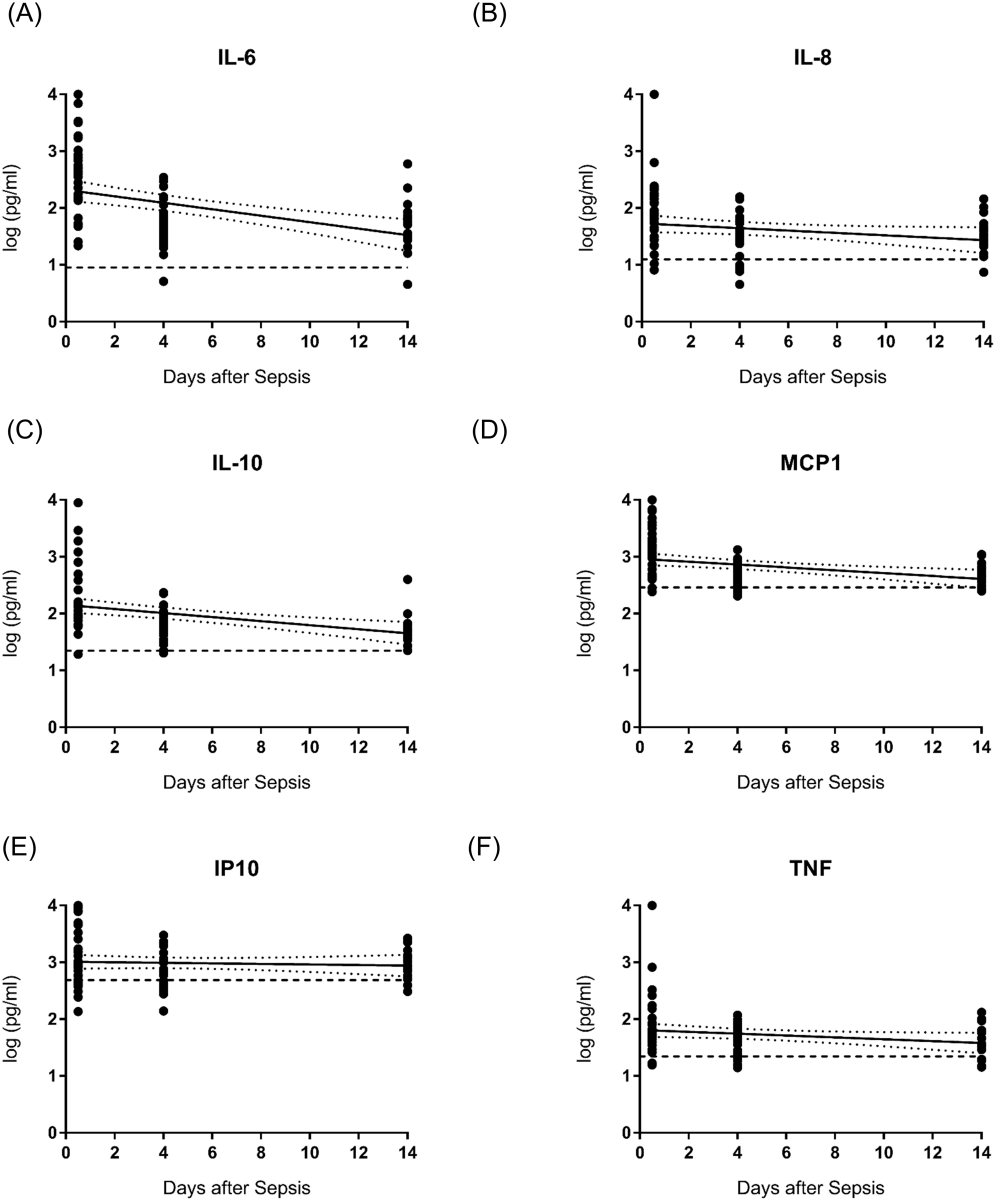
Cytokines IL-6 **(A)**, IL-10 **(C)**, and MCP-1 **(D)** levels had a significant, inverse correlation with days after sepsis (p<0.05), whereas IL-8 **(B)**, IP-10 **(E)**, and TNF **(F)** levels demonstrated a lack of significant correlation. Data are best-fit line with 95% confidence bands. Dashed line represents mean levels of healthy controls.

**Fig 3:**
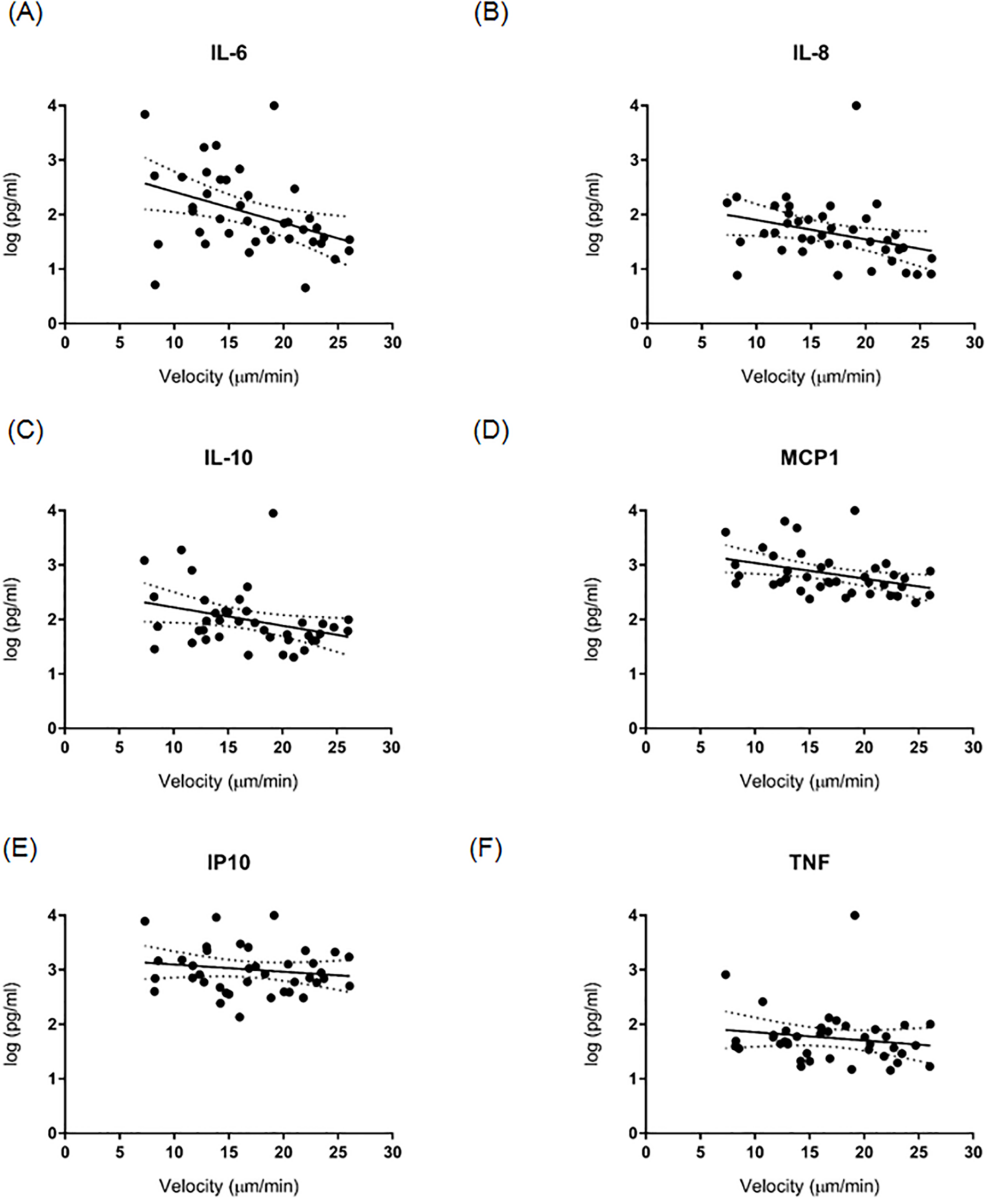
Cytokines IL-6 **(A)**, IL-8 **(B)**, IL-10 **(C)**, and MCP-1 **(D)** levels had a significant, inverse correlation with spontaneous neutrophil velocity (p<0.05), whereas IP-10 **(E)** and TNF **(F)** levels demonstrated a lack of significant correlation. Data are best-fit line with 95% confidence bands.

Circulating levels of the cytokines IL-6, IL-8, IL-10, MCP-1, IP-10, and TNF were significantly increased in septic patient at all three time points (<12 hours, 4 days, and 14 days after sepsis) compared to healthy controls. Concentrations of all cytokines were greatest at less than 12 hours post-sepsis at the same time spontaneous neutrophil migration was at its lowest, consistent with an early sepsis-induced ‘cytokine storm’. Regression analyses demonstrated significant, inverse correlations between IL-6, IL-10, and MCP-1 levels and days after sepsis (Fig 2A, 2C and 2D). There were no significant correlations between IL-8, IP-10, and TNF levels and days after sepsis (Fig 2B, 2E and 2F). There were significant, inverse correlations between IL-6, IL-8, IL-10, and MCP-1 levels and spontaneous neutrophil velocities (Fig 3A–3D). There were no significant correlations between IP-10 and TNF levels and spontaneous neutrophil velocities (Fig 3E and 3F).

## Discussion

Circulating neutrophils from septic patients had significant reduction in spontaneous neutrophil migration velocity compared to healthy control subjects. Using isolated neutrophils on a microfluidic device, Lu et al. previously demonstrated a reduction in neutrophil chemotaxis velocity to lipopolysaccharide in septic patients compared to healthy controls [19]. Here, using whole blood samples velocity in the absence of a chemoattractant, we demonstrated that spontaneous neutrophil migration is reduced the greatest <12 hours following sepsis protocol onset and improved with increasing number of days after sepsis. Neutrophil velocities of septic patients reached equivalent values of age- and gender- matched controls by 14 days after sepsis. Similar findings were seen in a previous study investigating the directional migration speed of isolated neutrophils following burn injury [20]. One explanation for the observed decrease in neutrophil migration abilities is that high levels of circulating proinflammatory cytokines early in sepsis increase levels of inducible nitric oxide which in turn may decrease neutrophil adhesion and transmigration to inflammatory sites [21, 22]. Additionally, it has been shown that there is a significant increase in rigidity of neutrophils isolated from septic patients compared to normal controls [23]. The surface expression of the chemokine (C-X-C motif) receptor 2 (CXCR2) and adhesion molecule CD11b have also been found to be reduced on neutrophils isolated from patients with septic shock compared with healthy controls [9]. Altogether, this may contribute to a decreased ability of neutrophils to properly migrate.

A key advantage of the current study over previous neutrophil studies in sepsis is the minimal amount of whole blood necessary for this assay and the ability to track individual neutrophils. Each microfluidic device is filled with just two microliters of whole blood stained with Hoechst. In addition to allowing smaller blood volumes, the use of whole blood preserves the in vivo physiological environment and circumvents the need for neutrophil isolation which may induce artificial stimulation. Likewise, the use of whole blood and absence of a chemoattractant makes the assay logistically simple.

We demonstrated for the first time a decrease in spontaneous neutrophil migration velocity among whole blood samples from septic adults. This may signify an impaired ability of neutrophils to respond to sites of infection during the acute phase of sepsis. The current study remains observational, and future studies should investigate stimulating human septic neutrophils *ex vivo* with immune agonists with the goal of augmenting spontaneous neutrophil migration velocity in early sepsis.

## Abbreviations

AKI: Acute kidney injury
ANOVA: Analysis of Variance
APACHE II: Acute Physiology And Chronic Health Evaluation II
ARDS: Acute Respiratory Distress Syndrome
BMI: Boyd Mass Index
CHF: Congestive Heart Failure
CXCR2: Chemokine (C-X-C motif) Receptor 2
EDTA: Ethylenediaminetetraacetic Acid
ESRD: End-stage Renal Disease
HBSS: Hank’s Balanced Salt Solution
HIV: Human Immunodeficiency Virus
ICU: Intensive Care Unit
IL: Interleukin
IP-10: Interferon-induced Protein-10
IRB: Institutional Review Board
KDIGO: Kidney Disease Improving Global Outcomes Staging
LOS: Length of Stay
MCP-1: Monocyte Chemotactic Protein-1
MEWS-SRS: Modified Early Warning Signs—Sepsis Recognition System
NSTI: Necrotizing Soft Tissue Infection
NYHA: New York Heart Association
SICU: Surgical Intensive Care Unit
SOFA: Sequential Organ Failure Assessment
TNF: Tumor Necrosis Factor
UTI: Urinary tract infection
WBLC: Whole Blood Loading Chamber

## References

[1] Angus DC, Linde-Zwirble WT, Lidicker J, Clermont G, Carcillo J, Pinsky MR. Epidemiology of severe sepsis in the United States: analysis of incidence, outcome, and associated costs of care. Critical care medicine. 2001;29(7):1303–10. Epub 2001/07/11. . 11445675 10.1097/00003246-200107000-00002

[2] Moore LJ, Moore FA, Todd SR, Jones SL, Turner KL, Bass BL. Sepsis in general surgery: the 2005–2007 national surgical quality improvement program perspective. Archives of surgery (Chicago, Ill: 1960). 2010;145(7):695–700. Epub 2010/07/21. 10.1001/archsurg.2010.107 . 20644134

[3] Wafaisade A, Lefering R, Bouillon B, Sakka SG, Thamm OC, Paffrath T, et al Epidemiology and risk factors of sepsis after multiple trauma: an analysis of 29,829 patients from the Trauma Registry of the German Society for Trauma Surgery. Critical care medicine. 2011;39(4):621–8. Epub 2011/01/19. 10.1097/CCM.0b013e318206d3df . 21242798

[4] Moore LJ, McKinley BA, Turner KL, Todd SR, Sucher JF, Valdivia A, et al The epidemiology of sepsis in general surgery patients. The Journal of trauma. 2011;70(3):672–80. Epub 2011/05/26. 10.1097/TA.0b013e31820e7803 . 21610358

[5] Benjamim CF, Ferreira SH, Cunha FQ. Role of nitric oxide in the failure of neutrophil migration in sepsis. The Journal of infectious diseases. 2000;182(1):214–23. Epub 2000/07/07. 10.1086/315682 . 10882600

[6] Alves-Filho JC, Benjamim C, Tavares-Murta BM, Cunha FQ. Failure of neutrophil migration toward infectious focus in severe sepsis: a critical event for the outcome of this syndrome. Memorias do Instituto Oswaldo Cruz. 2005;100 Suppl 1:223–6. Epub 2005/06/18 . 15962127 10.1590/s0074-02762005000900038

[7] Kurihara T, Jones CN, Yu YM, Fischman AJ, Watada S, Tompkins RG, et al Resolvin D2 restores neutrophil directionality and improves survival after burns. FASEB journal: official publication of the Federation of American Societies for Experimental Biology. 2013;27(6):2270–81. Epub 2013/02/23. 10.1096/fj.12-219519 . 23430978 PMC3659356

[8] Tavares-Murta BM, Zaparoli M, Ferreira RB, Silva-Vergara ML, Oliveira CH, Murta EF, et al Failure of neutrophil chemotactic function in septic patients. Critical care medicine. 2002;30(5):1056–61. Epub 2002/05/15. . 12006803 10.1097/00003246-200205000-00017

[9] Chishti AD, Shenton BK, Kirby JA, Baudouin SV. Neutrophil chemotaxis and receptor expression in clinical septic shock. Intensive care medicine. 2004;30(4):605–11. Epub 2004/03/03. 10.1007/s00134-004-2175-y . 14991094

[10] Arraes SM, Freitas MS, da Silva SV, de Paula Neto HA, Alves-Filho JC, Auxiliadora Martins M, et al Impaired neutrophil chemotaxis in sepsis associates with GRK expression and inhibition of actin assembly and tyrosine phosphorylation. Blood. 2006;108(9):2906–13. Epub 2006/07/20. 10.1182/blood-2006-05-024638 . 16849637

[11] Jones CN, Moore M, Dimisko L, Alexander A, Ibrahim A, Hassell BA, et al Spontaneous neutrophil migration patterns during sepsis after major burns. PLoS One. 2014;9(12):e114509 Epub 2014/12/10. 10.1371/journal.pone.0114509 . 25489947 PMC4260850

[12] Ellett F, Jorgensen J, Marand AL, Liu YM, Martinez MM, Sein V, et al Diagnosis of sepsis from a drop of blood by measurement of spontaneous neutrophil motility in a microfluidic assay. Nature Biomedical Engineering. 2018;2(4):207–14. 10.1038/s41551-018-0208-z PMC6166236 30283724

[13] Loftus TJ, Mira JC, Ozrazgat-Baslanti T, Ghita GL, Wang Z, Stortz JA, et al Sepsis and Critical Illness Research Center investigators: protocols and standard operating procedures for a prospective cohort study of sepsis in critically ill surgical patients. BMJ open. 2017;7(7):e015136 Epub 2017/08/03. 10.1136/bmjopen-2016-015136 . 28765125 PMC5642775

[14] Stortz JA, Murphy TJ, Raymond SL, Mira JC, Ungaro R, Dirain ML, et al Evidence for Persistent Immune Suppression in Patients Who Develop Chronic Critical Illness After Sepsis. Shock. 2018;49(3):249–58. Epub 2017/09/09. 10.1097/SHK.0000000000000981 . 28885387 PMC5809297

[15] Croft CA, Moore FA, Efron PA, Marker PS, Gabrielli A, Westhoff LS, et al Computer versus paper system for recognition and management of sepsis in surgical intensive care. J Trauma Acute Care Surg. 2014;76(2):311–7; discussion 8–9. 10.1097/TA.0000000000000121 . 24458039

[16] Levy MM, Fink MP, Marshall JC, Abraham E, Angus D, Cook D, et al 2001 SCCM/ESICM/ACCP/ATS/SIS International Sepsis Definitions Conference. Crit Care Med. 2003;31(4):1250–6. 10.1097/01.CCM.0000050454.01978.3B . 12682500

[17] Phua J, Ngerng W, See K, Tay C, Kiong T, Lim H, et al Characteristics and outcomes of culture-negative versus culture-positive severe sepsis. Crit Care. 2013;17(5):R202 10.1186/cc12896 . 24028771 PMC4057416

[18] Arena ET, Rueden CT, Hiner MC, Wang S, Yuan M, Eliceiri KW. Quantitating the cell: turning images into numbers with ImageJ. Wiley interdisciplinary reviews Developmental biology. 2017;6(2). Epub 2016/12/03. 10.1002/wdev.260 . 27911038

[19] Lu X, Lv C, Qu Y, Luo Y, Lin B, Zhan L, et al Sepsis-induced impairment of neutrophil chemotaxis on a microfluidic chip. Immunology letters. 2016;173:55–60. Epub 2016/03/27. 10.1016/j.imlet.2016.03.010 . 27016001

[20] Butler KL, Ambravaneswaran V, Agrawal N, Bilodeau M, Toner M, Tompkins RG, et al Burn injury reduces neutrophil directional migration speed in microfluidic devices. PloS one. 2010;5(7):e11921 Epub 2010/08/07. 10.1371/journal.pone.0011921 . 20689600 PMC2912851

[21] Alves-Filho JC, Spiller F, Cunha FQ. Neutrophil paralysis in sepsis. Shock (Augusta, Ga). 2010;34 Suppl 1:15–21. Epub 2010/08/28. 10.1097/SHK.0b013e3181e7e61b . 20714263

[22] Zhang F, Liu AL, Gao S, Ma S, Guo SB. Neutrophil Dysfunction in Sepsis. Chinese medical journal. 2016;129(22):2741–4. Epub 2016/11/09. 10.4103/0366-6999.193447 . 27824008 PMC5126167

[23] Drost EM, Kassabian G, Meiselman HJ, Gelmont D, Fisher TC. Increased rigidity and priming of polymorphonuclear leukocytes in sepsis. American journal of respiratory and critical care medicine. 1999;159(6):1696–702. Epub 1999/06/03. 10.1164/ajrccm.159.6.9803061 . 10351906

